# “There’s plenty of room at the bottom”: deep brain imaging with holographic endo-microscopy

**DOI:** 10.1117/1.NPh.11.S1.S11504

**Published:** 2024-01-19

**Authors:** Hana Uhlířová, Miroslav Stibůrek, Tomáš Pikálek, André Gomes, Sergey Turtaev, Petra Kolbábková, Tomáš Čižmár

**Affiliations:** a Institute of Scientific Instruments of the Czech Academy of Sciences, Brno, Czech Republic; b Leibniz Institute of Photonic Technology, Jena, Germany; c Friedrich Schiller University Jena, Institute of Applied Optics, Jena, Germany

**Keywords:** complex media photonics, wavefront shaping, endoscope, multimode fiber, in-vivo imaging, deep brain imaging

## Abstract

**Significance:**

Over more than 300 years, microscopic imaging keeps providing fundamental insights into the mechanisms of living organisms. Seeing microscopic structures beyond the reach of free-space light-based microscopy, however, requires dissection of the tissue—an intervention seriously disturbing its physiological functions. The hunt for low-invasiveness tools has led a growing community of physicists and engineers into the realm of complex media photonics. One of its activities represents exploiting multimode optical fibers (MMFs) as ultra-thin endoscopic probes. Employing wavefront shaping, these tools only recently facilitated the first peeks at cells and their sub-cellular compartments at the bottom of the mouse brain with the impact of micro-scale tissue damage.

**Aim:**

Here, we aim to highlight advances in MMF-based holographic endo-microscopy facilitating microscopic imaging throughout the whole depth of the mouse brain.

**Approach:**

We summarize the important technical and methodological prerequisites for stabile high-resolution imaging *in vivo*.

**Results:**

We showcase images of the microscopic building blocks of brain tissue, including neurons, neuronal processes, vessels, intracellular calcium signaling, and red blood cell velocity in individual vessels.

**Conclusions:**

This perspective article helps to understand the complexity behind the technology of holographic endo-microscopy, summarizes its recent advances and challenges, and stimulates the mind of the reader for further exploitation of this tool in the neuroscience research.

## 1 Introduction

A great effort has been devoted in recent years to developing techniques and tools that would enable imaging deep into the living tissue. The illumination intensity is fundamentally limited by scattering and absorption in the tissue, exponentially decreasing with depth. One of the strategies to reach deeper is extending the wavelength of illumination to optical windows around 1300, 1700, and 2200 nm exploiting multiphoton excitation. At 1700 nm, fluorescently labeled vasculature can be imaged down to 2.1 mm, which currently represents the experimental depth limit for imaging in the brain achieved by 3-photon excitation.^1^ For depths greater than that, endoscopes that exploit optical elements relaying light to the location of interest and back are used. There are few types of endoscopic probes, graded-index (GRIN) lenses being arguably the most prominent ones used for *in-vivo* brain imaging. The diameters of GRIN lenses reach anywhere from a few hundreds of micrometers to a few millimeters. The millimeter-sized lenses, typically doublets or triplets, allow for correction of optical aberrations providing high-quality images with sub-cellular resolution.^2^ Embedding these implants is, however, conditioned by high-impact surgeries extracting the soft tissue above the target area. Decreasing the diameter of the lenses lowers the impact on the tissue, yet any aberration correction becomes increasingly difficult.^3^ A promising idea of aberration correction in a thin (350  μm) GRIN lens has been presented recently,^4^ where a 3D-printed correction lens was applied at the input facet, yielding sub-micron resolution across a spherically curved focal plane (FP) at the distal end. Image data acquired here from a curved space may be challenging to interpret, especially in regions with strong laminar organization. An alternative concept of endoscopy, which has recently found its way into the realm of *in-vivo* brain imaging, exploits multimode optical fibers (MMFs).^5^^–^^8^ It builds on wavefront shaping technology to characterize the light transport through the system and subsequently generate a desired optical field behind the MMF. For imaging, the sample in proximity of the distal end of the fiber is typically exposed to a sequence of diffraction-limited foci organized across an orthogonal grid, thereby mimicking raster-scanning. For each focus position, the emitted fluorescence signal is simultaneously collected backward by the same fiber and, after spectral separation from the excitation light, its intensity is measured by a sensitive detector. In MMFs, aberration-free diffraction-limited resolution can be achieved across the whole planar field of view (FOV), regardless of the fiber footprint and length, facilitating by far the most atraumatic imaging at unprecedented depths. In this perspective article, we summarize the latest technological and methodological advancements that enable sub-micron structural and functional *in-vivo* imaging throughout the whole depth of the mouse brain and showcase records of principal brain tissue compartments and their function.

## 2 Working Principle

**Fig. 1:**
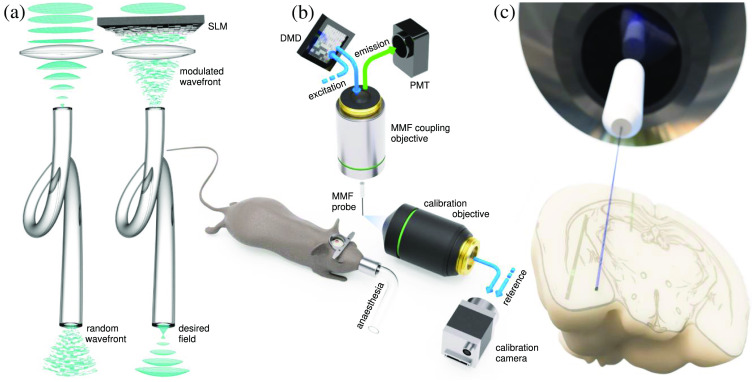
Principle of MMF-based holographic endoscopy: (a) light coupled into an MMF is split into a variety of propagating modes, which then present different phase delays at the output, yielding a random wavefront (left). An SLM can be used to modulate the input wavefront, creating a desired filed at the output (right). (b) Modulated illumination diffracted off the DMD couples into the MMF and the output is imaged on the camera while interfering with a reference beam. Upon calibration, the calibration module is withdrawn, and the animal model is inserted. (c) The MMF probe embedded in a ferrule in proportion to the mouse brain (brain model from Allen Reference Atlas—Adult Mouse^9^). Panels (b) and (c) adapted from Ref. 8.

Light propagating through an MMF is coupled into a multitude of modes, which scrambles its wavefront and polarization yielding a speckle pattern at the fiber output [Fig. 1(a) left]. The transport of light through MMF is, however, an almost perfectly linear and deterministic process, which can be controlled using, e.g., a spatial light modulator [SLM, Fig. 1(a) right]. Digital micro-mirror devices (DMDs) present currently the most convenient tools for such wavefront control in single-photon imaging applications. In the off-axis regime, they can be used for both amplitude and phase manipulation of light with high fidelity,^10^ creating any desired electromagnetic field at the fiber output within the constraints given by the fiber dimensions and NA. Importantly, their refresh rate allows for fast scanning of *in-vivo* fluorescence dynamics.

A detailed description of a holographic endoscope setup can be found e.g., in Ref. 7. Briefly, the modulated light diffracted off the DMD is coupled into the MMF probe, and the output is imaged using a camera while it interferes with a reference wave [Fig. 1(b)]. This way, a complex linear operator between the input and output is measured, which is typically referred to as the transmission matrix (TM). The TM, measured in both polarization states, prescribes the appropriate configuration of the DMD micro-mirrors that generates the desired field at the output. In applications relying on low photon numbers, such as *in-vivo* fluorescence imaging, the optimal pattern for illuminating the sample is a diffraction-limited focal point.^11^ Upon the calibration step, the calibration module is withdrawn, and the probe is inserted into the tissue. The focal point is sequentially projected in a rectangular grid pattern and the excited fluorescence from the sample is collected back through the same fiber and detected using most commonly the photo-multiplier tube. The road from assembling a holographic endoscope setup to reliable *in-vivo* imaging is, however, not a direct one. In the next section, we describe the main technological and methodological advancements that have made this leap possible.

## 3 Major Technological and Methodological Prerequisites for High-Quality *In-Vivo* Imaging

### 3.1 Thermal Stability of the DMD

**Fig. 2:**
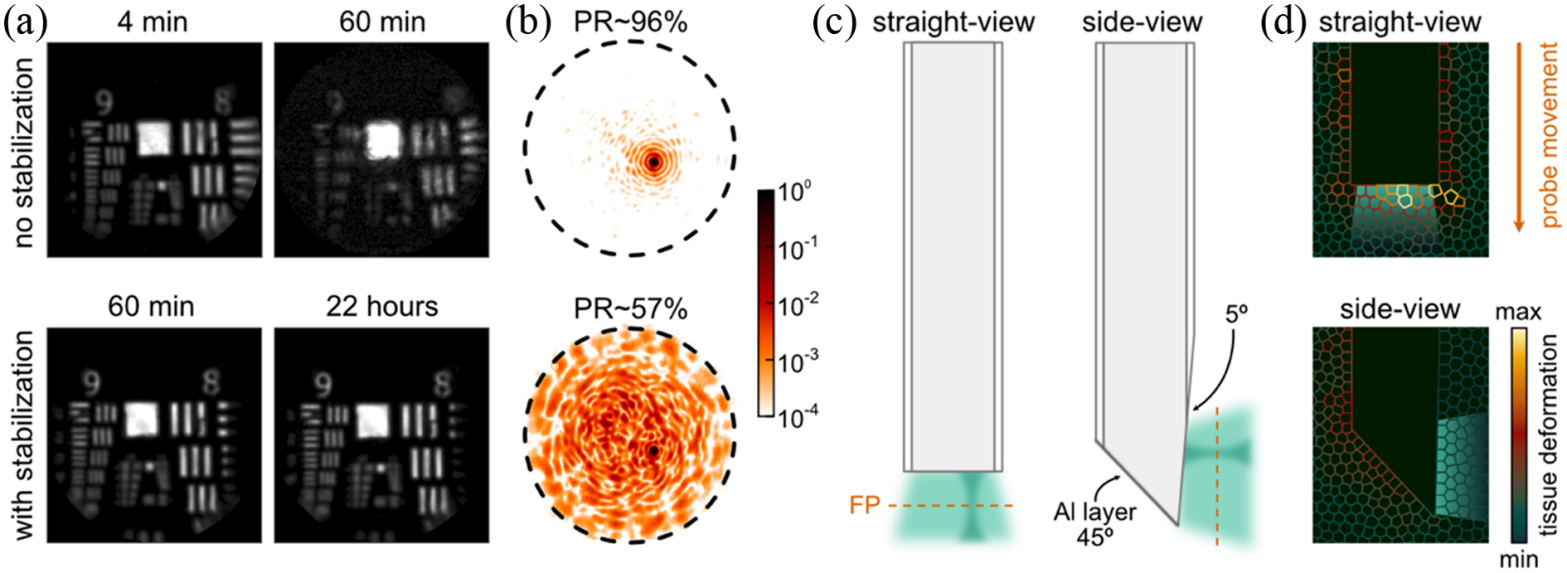
Technological advancements: (a) thermal stability of the DMD allows long-term high-quality imaging: a fluorescence USAF target imaged without (top) and with (bottom) the temperature stabilization of the DMD 4 min, 60 min, and 22 h after the calibration (adapted with permission from Ref. 12 © Optica). (b) Focal point at the output of the fiber with PR∼96% (top) and PR∼57% (bottom). (c) The FP in the conventional “straight-view” configuration lies below the output facet of the fiber (left). In the “side-view” configuration, the distal end is polished to 45 deg and coated with aluminum (Al). The light is reflected off the mirror, exits the probe through a window polished under a small angle on the side (∼5  deg), and forms the FP along the optical axis of the fiber (right). (d) Simulation of tissue deformation by insertion of the straight-view (top) and side-view (bottom) probe into an elastic medium divided into cell units. The color indicates the cell’s displacement due to the probe insertion (adapted with permission from Ref. 13 © Optica).

The TM is highly sensitive to any perturbations of the wavefront induced by mechanical or thermal changes in the light path. Any changes to the TM reduce the fidelity of light control at the tip of the fiber leading to degradation of the image quality. Therefore, one of the main prerequisites for *in-vivo* holographic endo-microscopy is stability. For example, the conformation of the step-index MMF used must remain the same during calibration and imaging. Furthermore, the wavefront at the input to the MMF must also be time-invariant. For commercially available step-index fibers of length up to ∼3  cm [Fig. 1(c)], any conformational changes can be avoided by careful control of the animal position during probe insertion, in which the probe remains stationary, and the animal is moved using stepper motors with feedback. The illumination wavefront, however, can deviate quite dramatically during imaging. The major source for such drift is related to the thermal load of the DMD induced by operation at the highest frame rates. This effect degrades the focal point over time and hence the quality of the acquired images, which would enforce repeated re-calibrations [Fig. 2(a) top]. Actively controlling and stabilizing the temperature of the DMD with a thermoelectric cooler preserves the input wavefront, facilitating high-quality imaging for tens of hours upon single calibration [Fig. 2(a) bottom].^12^

### 3.2 Maximizing the Imaging Capacity

#### 3.2.1 Optimizing the point spread function

The quality of a scanned image is determined by the point spread function (PSF) of the imaging system. The PSF of the holographic endo-microscope takes the shape of a diffraction-limited focus contaminated by certain level of undesired speckled background [Fig. 2(b)]. The fraction of power carried in the focus in respect to the total power leaving the fiber—referred to as the power ratio (PR)—represents a metric of focusing fidelity, dictating the contrast and hence the resolution of the image. Simulations show that, in fibers supporting more than 1000 optical modes, a precise complex control of the input optical field leads to a PSF with PR>99%.^14^ Practically, this requires (1) precise control of both orthogonal input polarization states, (2) optimization of the carrier frequencies of holograms projected on the DMD to avoid light from higher diffraction orders coupling into the fiber, (3) calibration with aberration-corrected wavefronts, and (4) complex modulation of light, i.e., control over both amplitude and phase. Under ideal experimental conditions, PRs as high as >96% can be reached [Fig. 2(b) top].^15^ In our imaging set-up, implementing the phase and polarization control, we routinely reach PR>55% [Fig. 2(b) bottom], which for us has represented a reasonable trade-off between the image quality and the complexity of the set-up.

#### 3.2.2 Resolution, field of view, and scanning speed

In the strive for increasing the image capacity, we implemented a remote, highly parallelized GPU computation which allowed us to use fibers with higher NA and core diameter. Specifically, we used fibers with NA of 0.37 and core diameter of 100  μm (CeramOptec Optran Ultra WFGE, cladding diameter 110  μm), supporting ∼28,000 modes. This enabled imaging structures with resolution of 0.8  μm in a FOV of ∼100  μm in diameter. Further increasing of resolution using higher NA fibers^16^^,^^17^ was not desirable due to the current limitations of the DMD’s onboard memory. Also increasing the FOV would trade off for resolution to keep the TM size optimal. The scanning speed is dictated by the DMD’s refresh rate (27 kHz), leading to scanning rates of 0.3 Hz for the 100  μm-wide FOV. Importantly, holographic manipulation of light inherently enables random-access scanning. This easily allows the implementation of distinct scanning schemes, e.g., along custom-designed trajectories or smaller areas/FOVs, thus boosting the scanning rate to up to a few kHz.

#### 3.2.3 Numerical re-focusing

The TM fully describes the light propagation through the optical system. Although the TM is recorded at a certain distance from the output facet (FP), it can be numerically propagated to a different distance allowing for re-focusing of the image. Volumetric imaging can therefore be acquired without any physical movement of the probe in the tissue—another feature that minimizes tissue disruption.

### 3.3 Side-View

A typical regime for imaging through the MMFs is a “straight-view,” looking directly below the output facet of the fiber [Fig. 2(c) left]. However, this region of tissue is affected the most by vertical movement of the probe, deforming and displacing the cells under inspection [Fig. 2(d) top]. A far superior solution turns out to be a “side-view” mode, in which the FP is parallel to the fiber optical axis. Such mode requires an MMF probe with its distal end polished to 45 deg and coated with a reflective aluminum layer [Fig. 2(c) right].^13^ The light propagated through the fiber is reflected off the mirror and exits the fiber through an optical window sideways. This way, the imaged region at the FP is unperturbed by movement of the probe [Fig. 2(d) bottom].^13^ All surfaces of the probe exposed to the tissue are further coated with a thin layer (∼2  μm) of parylene-C. This hydrophobic polymer ensures biocompatibility and further facilitates smooth movement of the probe in regards to the tissue.

## 4 Results

**Fig. 3:**
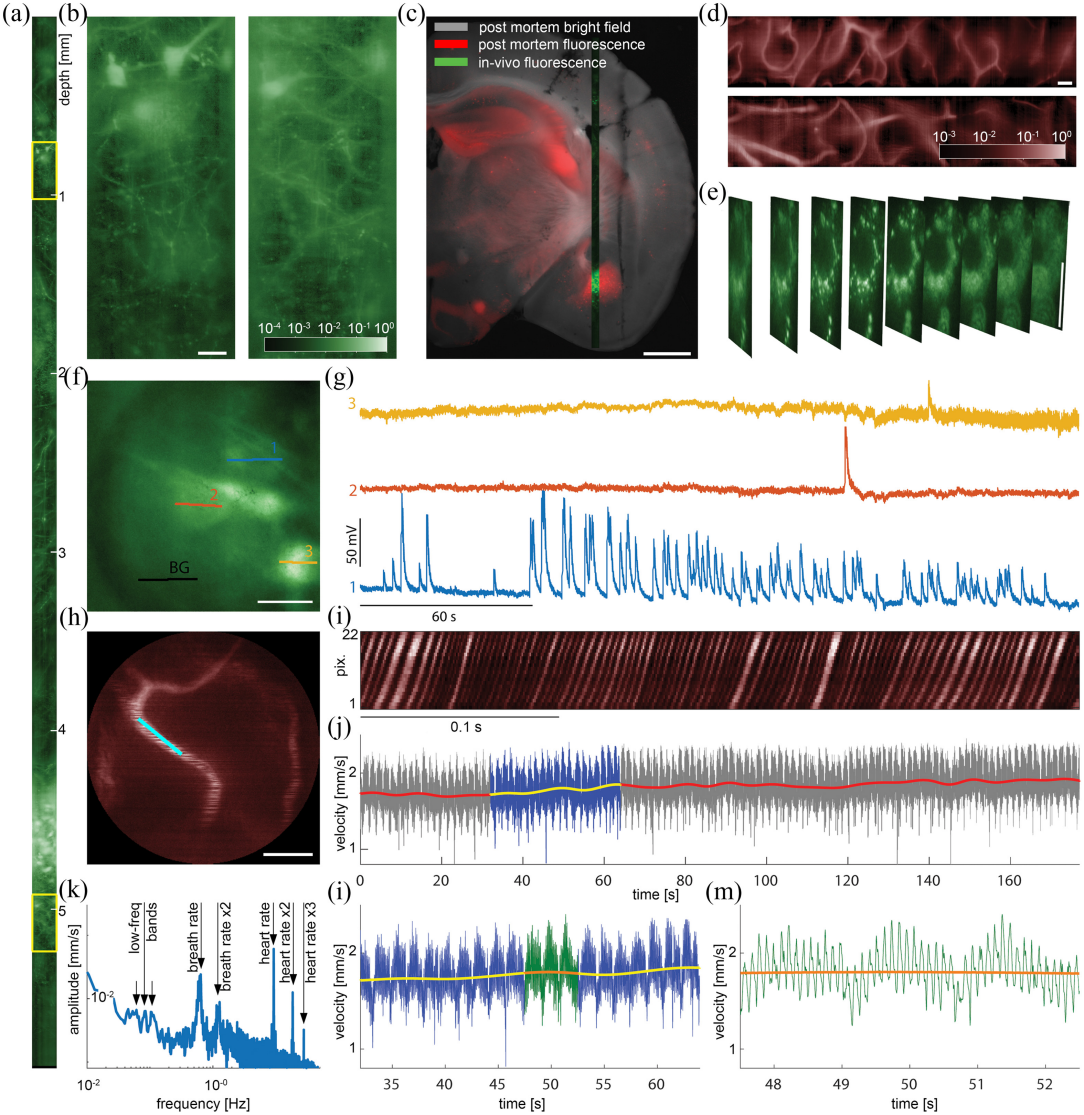
*In-vivo* structural and functional imaging: (a) an extended FOV captured along the whole fiber track from the top part of cortex all the way down below amygdala. (b) Zoomed-in sections from the yellow-framed regions in (a) showing sub-cellular features in detail. (c) A thin brain post-mortem section showing the overall GFP expression (red) in the wide-field image (gray) and the high-resolution GFP signal imaged *in vivo* through the MMF-probe along its track (green). (d) Extended FOVs of fluorescently labeled vessels acquired along the fiber track (images rotated by 90 deg). (e) A cholinergic neurone expressing ZsGreen captured at depth of ∼3.5  mm in different FPs exploiting the numerical refocusing. (f) A reference image of excitatory neurons expressing GCaMP with custom-defined scanning trajectories. (g) Calcium traces after subtraction of the background (BG) signal. (h) A reference image of fluorescently labeled vessels with the scanning trajectory (cyan). (i) Intensity profile along the scanning trajectory in time. (j), (l)–(m) Reconstructions of RBCs velocity trace and its zoomed-in sections. (k) Velocity power spectrum. The scale bars in (b), (d)–(f), and (h) correspond to the length of to 20  μm. The scale bar in (c) corresponds the length to 1 mm. Figure adapted from Ref. 8.

Implementation of the advancements described in the previous section allowed us to exploit this technology in routine endo-microscopy of brain structure and function^8^ *in vivo*. All animal experiments were conducted in accordance with protocols approved by the Branch Commission for Animal Welfare of the Ministry of Agriculture of the Czech Republic (permission nos. 47/2020 and 49/2020) limiting individual imaging sessions to 5 h. Imaging with the “side-view” probe facilitates smooth movement of the tissue in respect to the probe being immersed. In this regime, individual images acquired sequentially along the path can be stitched together during post-processing into an extended FOV spanning across the whole brain depth [Figs. 3(a)–3(c)]. The entire area displays homogeneous resolution appropriate for resolving sub-cellular features [Fig. 3(b)] and single vessels [Fig. 3(d)]. Using numerical refocusing, each scene can be captured in multiple focal planes [Fig. 3(e)] reconstructing volumes of up to ∼100×20×6000  μm.^3^

Strong tissue deformations and inevitable disruption of cellular connections induced by insertion of endoscopic probes yield deep-brain functional imaging highly challenging. Minimization of the footprint is therefore of high importance, especially when investigating processes dependent on long-range signaling. To this end, we demonstrated intracellular calcium imaging of GCaMP-expressing excitatory neurons [Figs. 3(f)–3(g)], sampling multiple regions of interest with custom-defined scanning trajectories at tens of Hz. In another example, we showcase imaging of blood flow in single vessels [Figs. 3(h)–3(m)] at a scanning speed of ∼1000  Hz. Using the intensity contrast between the dark red blood cells (RBCs) and fluorescently labeled plasma, trajectories of single cells along the scan can be recorded and used to calculate the RBC speed time-course [Figs. 3(j), 3(l)–3(m)] and its power spectrum [Fig. 3(k)], analogously to a well-established method used in free-space optical microscopy.^18^

## 5 Discussion

In the last two decades, the field of neuroscience has witnessed a transformative era with the emergence of novel *in-vivo* optical and molecular tools. These advancements have empowered researchers to conduct detailed studies of neuronal circuits coding for sensory integration, perception, memory, behavior and also the tightly coupled vascular and hemodynamic function fueling the circuitry. The imperative to comprehend the mechanisms underlying these circuits has been driven by a keen interest in advancing our understanding of brain diseases. Microscopic technology, pivotal in this quest, has been spurred to image more, faster, deeper, with greater detail and ideally in naturally behaving animals. No single existing technology fulfils all these criteria.

Wide-field microscopes exploiting single photon (1P) excitation allow for fast imaging of superficial signals across tens of ${\mathrm{mm}}^{2}$. Their resolution is mediocre (few microns to tens of microns) due to light scattering and a lack of depth discrimination. 1P microscopes have been successfully miniaturized into open platforms providing simple and low-cost tools for mesoscopic imaging in behaving animals. Both modalities have been exploited with advantage in studies of cortex-wide representation of sensory-evoked responses, open field and social behaviors,^19^ sleep,^20^ hippocampal memory encoding,^21^ activity mapping,^22^ vasculature,^23^ and hemodynamics.^24^^,^^25^

When higher resolution and imaging deeper in the tissue is desired, typically multiphoton excitation microscopy is the technique of choice. It provides sub-micron lateral resolution and few-micron axial resolution owing to the localized excitation. Imaging of fine structures such as dendritic spines and axonal terminals is feasible, although at the cost of limited speed by scanning. Many strategies have pushed this technology to its resolution and speed limits including beam shaping,^26^^–^^29^ temporal focusing,^30^ adaptive optics,^31^^,^^32^ spatiotemporal multiplexing,^33^ and non-degenerate microscopy,^34^ just to name a few. Also, multiphoton microscopes have been miniaturized to facilitate single-cell^35^^–^^37^ or even single-spine^37^ imaging in behaving animals. Both single-photon and multiphoton excitation imaging can be relayed to deeper brain structures using GRIN lenses implants.^21^^,^^38^ Preserving sub-cellular resolution with implants is very challenging^3^ and often requires bulky micro-objectives forcing invasive interventions including removal of the tissue above the target location. This may be one of the main reasons why majority of deep-brain high-resolution micro-endoscopy is exploited in dorsal hippocampus at the deepest.^2^^,^^3^^,^^29^^,^^39^^,^^40^ On the other side of the implants’ invasiveness spectrum are ultra-thin fibers used in photometric measurements.^41^^–^^44^ Here, the specificity is provided only by the promoter-encoded expression of the fluorescent indicator. Hence, interpretation of functional data may be difficult due to artifacts resulting from movement of the probe within the tissue, autofluorescence, or absorption changes induced by hemodynamics.^41^^,^^45^^–^^47^ Studies of brain regions located below the dorsal hippocampus, which would benefit from spatially encoded information, can now be addressed using MMF-based holographic endoscopes. Structures can be resolved with sub-micron resolution, although scattering in densely labeled samples may be a limiting factor inherently to the 1P excitation process. The maximum FOV is determined by the fiber core diameter, capturing typically few cell bodies and the surrounding processes. The scanning frequency is limited by the refresh rate of the DMD and is currently sufficient for calcium and RBC imaging in few tens to thousands of pixels. Finally, currently available MMFs do not allow for imaging under changing fiber contortions and therefore experiments are confined to head-restrained animals. Despite these limiting factors, holographic endoscopy represents a unique tool for deep-brain imaging and yet another color to the palette of tools facilitating across-scale studies of the brain.

## 6 Conclusion and Outlook

Recent technological and methodological advancements in holographic endo-microscopy facilitated, for the first time, acquisition of diffraction-limited fluorescence microscopic records of cells and vessels *in vivo* throughout the whole brain depth. Fast scanning along custom-designed trajectories has been used to record single-cell-resolved intracellular calcium and single-vessel blood flow. Recording the structures of interest with high resolution rather than generic intensity provided by fiber photometry—although cell-type specific—represents an important step toward understanding the origin of the detected signal. Ambiguities, such as somatic versus dendritic signaling, correction of motion or hemodynamic artifacts or structure-specific signal variations, may now be addressed. Sampling a small FOV determined by the footprint of the probe can be limiting when it comes to testing of biological hypotheses. This limitation can be mitigated to some extent in post-processing by stitching of neighboring images acquired along the fiber track, which can span the whole brain depth.

The presented imaging modes have been achieved close to the limits of currently available technologies. The on-board memory of the DMD limits the number of patterns that can be uploaded in advance and then projected at the maximum speed corresponding to the DMD’s refresh rate. The DMD’s memory hence restricts the maximum dimensions of the TM determining the image capacity, i.e., the FOV and resolution. Future generations of DMDs that may support on-the-fly control would allow sequencing an unlimited number of patters at the maximum speed. With such technology, the only constrain to consider would be the trade-off between the number of image pixels and the impact of the probe footprint.

While the captured intracellular calcium dynamics and RBC velocity represent functional footprints of the brain in action, all measurements shown here have been carried out in anesthetized animals. Although many important questions can be addressed in head-restrained animals, anesthesia presents confounding effects that are difficult to account for. An inevitable next step therefore represents transferring this technology into awake animal models. No technological obstacles should prevent this leap, provided the animal is head-fixed. In freely moving animals, the major challenge comes with changing contortions of the fiber, which in conventional MMFs induce changes of the TM. Adoption of new strategies exploiting optimal fibers,^48^ powerful algorithms utilizing pre-calibrated TMs in multiple conformations,^49^ and exploiting fiber-specific memory effects^50^ are most likely going to facilitate the first prove of principle experiments soon.

An important advancement of holographic endo-microscopy would certainly be in the direction of out-of-focus light rejection. Although the basic principles of confocal and multiphoton microscopy through the MMFs have been demonstrated,^51^^,^^52^ their application in fluorescence imaging is waiting for new and practical concepts. Despite the miniature form factor of the MMF, functional data need to be carefully evaluated for artifacts induced by mechanical stimulation of the tissue by the probe. Occasionally, the insertion of the fiber can cause bleeding at the imaging site, which can be seen immediately. In calcium imaging, insertion of the probe may mechanically stimulate the neurons leading to an irreversible increase of intracellular calcium. Such data should be excluded from the particular study. This issue may be addressed in the future by chronically implanted connector solutions, preventing physical contact of the moving probe with the tissue. Such solutions could also facilitate repeated imaging of the same FOV in remote intervals of time.

In conclusion, the prospect of holographic endo-microscopy in awake behaving animals paves the way to exploring the ventral realm of their brain. Many important questions involving physiological and pathological neuromodulations, functional connections in deep brain nuclei, deep-brain stimulation related circuitry, sub-cortical single-vessel hemodynamics, or neuro-vascular and astro-vascular coupling now remaining unexplored are slowly becoming within fiber-reach.

## Data Availability

All presented data and codes can be accessed through repositories indicated in the referenced original publications.
